# Different expression of B7-H3 in the caput, corpus, and cauda of the epididymis in mouse

**DOI:** 10.1186/s12894-017-0215-5

**Published:** 2017-04-04

**Authors:** Kai Li, Xuedong Wei, Guangbo Zhang, Miao Li, Xuefeng Zhang, Chenhao Zhou, Jianquan Hou, Hexing Yuan

**Affiliations:** 1 Department of Urology, The First Affiliated Hospital of Soochow University, NO.188 Shizi Road, Suzhou, 215006 Jiangsu China; 2 The Institute of Clinical Immunology, The First Affiliated Hospital of Soochow University, NO. 708 Renmin Road, Suzhou, 215006 China

**Keywords:** B7-H3, Caput, Corpus, Cauda, Epididymis, Sperm maturation, Mouse

## Abstract

**Background:**

B7-H3, a member of the B7 family of the Ig superfamily of proteins, has been detected in the epididymis, which is a storage organ related to sperm maturation. However, the characteristics of its expression in different regions of the epididymis remain unknown. Our aim was to investigate the expression of B7-H3 in the caput, corpus, and cauda of the epididymis.

**Methods:**

We extracted epididymis specimens from adult male C57BL/6 mice. The expression of B7-H3 was then measured with immunohistochemistry, enzyme-linked immunosorbent assay (ELISA) and western blotting.

**Results:**

B7-H3 protein was predominantly detected on the membrane and in the cytoplasm of the principal cells in the epididymis. Moreover, the level of B7-H3 in the corpus of the mouse epididymis was significantly higher than that in the caput (*p* < 0.05) or the cauda of the epididymis (*P* < 0.05). However, there was no remarkable difference in the level of B7-H3 between the caput and the cauda (*p* > 0.05).

**Conclusions:**

The caput, corpus, and cauda of the mouse epididymis all expressed B7-H3 protein. However, the levels of B7-H3 were different in the three regions of the epididymis.

## Background

The epididymis is a storage organ of sperm and is related to male fertility. From proximal to distal, the epididymis is divided into caput, corpus, and cauda regions. This conventional division has been widely used in studies on the biological functions of the epididymis, including studies of gene expression, protein secretion, and many aspects of sperm maturation [1]. The epididymis provides a specific environment which plays an essential role in promoting the final maturation of sperm [2]. It can take sperm a few days to weeks to travel throughout the epididymis. During this time almost all of sperm gradually acquire natural fertilizing ability – including progressive motility, the ability to undergo capacitation and hyperactivation [3]. The epididymal epithelium has the functions of secretion and absorption. At least 66% of identified proteins can be secreted into the epididymal fluid [4]. A series of interactions between sperm and some specific proteins present in the epididymal fluid are believed to contribute to the final maturation of sperm [5].

B7-H3, also known as CD276, a novel member of the B7 immunoregulatory family, was initially identified from a human dendritic cell cDNA library in 2001 [6]. It is a 316-amino acid (aa) type I transmembrane glycoprotein [7]. This protein also exists in a soluble form. A study by our group reported that soluble B7-H3 could be detected in expressed prostatic secretions of the healthy donors and patients with chronic prostatitis [8]. Several studies have indicated that B7-H3 protein is expressed in dendritic cells, the heart and kidneys [9] as well as genital tissues, such as prostate, testis, and epididymis [10]. In the current study, B7-H3 is mainly thought to have a significant role in the immune system. Initial experiments reported that B7-H3 might upregulate T cell activation [10] and promote T cell proliferation, as well as cytokine production [9, 11]. However, some subsequent studies showed that B7-H3 had a negative regulatory function in T cell -mediated immune responses both in vitro and in vivo [12, 13]. Moreover, some recent reports provided novel insights into the nonimmunological role of B7-H3 protein. Sun et al. suggested that B7-H3 could inhibit the expression of vascular endothelial growth factor (VEGF) [14], while Xu et al. found that the stimulation of B7-H3 could promote the differentiation of human marrow stromal cells (hMSCs) into osteoblasts [15]. Furthermore, B7-H3 is also expressed in fibroblast-like synoviocytes and other fibroblasts [16]. Consequently, the biological functions of B7-H3 remain unclear.

Suh et al. discovered that B7-H3 was expressed in the epididymis [10]. Additionally, a previous study by our group showed that in vitro, B7-H3 could promote sperm progressive motility [17]. Due to these findings, we focused on: (1) whether B7-H3 is expressed in all regions of the mouse epididymis, and (2) whether the levels of B7-H3 expressed in the caput, corpus, and cauda of the mouse epididymis are different.

## Methods

### Animals and samples collection

**Fig. 1:**
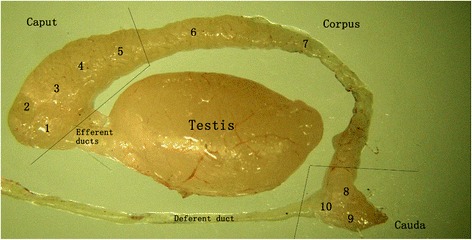
Mouse testis and epididymis. The epididymis was subdivided into 10 segments (1–10): 1–5, caput; 6–7, corpus; 8–10, cauda

The experimental protocol was approved by the animal ethics committee of Soochow University. All procedures in the project were executed based on the guidelines for animal experiments. Male C57BL/6 mice for this research were purchased from Suzhou sealop match Biological Technology Co. Ltd. All of these mice were at ten weeks of age. All mice were kept in mesh bottom cages, and the room temperature was maintained at 22–25 °C. All mice had free access to water and food. The epididymis tissues were obtained fresh from twelve mice, which were randomly divided into three groups according to Johnston et al.[18]. Each epididymis was then segmented into caput, corpus, and cauda regions for ELISA (Fig. 1). Samples from ten mice were used in western blotting for each independent experiment. For immunohistochemical staining three mice had their epididymides extracted.

### Immunohistochemistry

The protocol of immunohistochemical staining was performed according to Li et al. [19]. Briefly, the paraffin -embedded epididymis tissues were cut into sections of 4 μm. The sections were dewaxed in xylene and rehydrated in a graded dilution of ethanol solutions, followed by heating the sections at 100 °C for 30 min in citrate solution. Endogenous peroxidase activity was blocked with 0.3% hydrogen peroxide for 20 min and washed with phosphate–buffered saline(PBS). Then, these sections were blocked with 5% BSA and incubated with B7-H3 antibody (dilution 1:200, Proteintech, China), overnight at 4 °C. These sections were then washed with PBS, and horseradish peroxidase -conjugated goat anti -rabbit IgG antibody was used as secondary antibody. Finally, the sections were counterstained with hematoxylin.

#### ELISA

During preparation, each tissue sample was collected, divided into three regions (Fig. 1) and weighted using the same method. Each region was cut into pieces of 1 mm^3^ and digested with IV collagenase for about 1 h (hour) at 37 °C. Then, the samples were prepared with PBS and homogenized in cell lysis buffer containing a protease inhibitor produced at our laboratory. After incubation on ice for 30 min (minutes), the homogenate was centrifuged at 14000 X gravity for 20 min at 4 °C and the supernatant was collected for ELISA assay. We used enzyme-linked immunosorbent assay kits, produced by Suzhou Bi Liya Biotechnology Co. Ltd, to measure B7-H3 in the caput, corpus, and cauda of the epididymis. These assay kits are specific for mouse B7-H3 at 1.0 ng/ml minimum detectable dose. The procedures were performed as recommended by the manufacturer. Assays were read at 450 nm in a Thermo Multiskan Mk3 microplate reader (Labsystems, Helsinki, Finland). We used a standard curve to determine B7-H3 concentration.

### Western blotting

Ten mice for each independent experiment were randomly selected for Western blotting. All epididymis tissues were dissected and homogenized in cell lysis buffer containing protease inhibitors. After homogenization and centrifugation, the supernatant was collected for Western blotting. The proteins were then extracted in 6 x SDS–PAGE sample loading buffer. The proteins were then separated by 10% SDS-PAGE and transferred to a 0.45 mm PVDF membrane for approximately 300 mA for 90 min. After transfer, the membrane was blocked in 5% fat-free milk/1xTBS/0.1%Tween for 1 h at room temperature, and incubated with B7-H3 antibody (dilution 1:1000, Lianke, China) and GAPDH antibody (dilution 1:1000, Sigma) overnight at 4 °C. The membrane was then incubated with the secondary antibody of horseradish peroxidase -conjugated goat anti-rabbit IgG antibody (dilution 1:10,000, Lianke, China) for 1 h at room temperature. The blots were visualized using the BeyoECL Plus substrate system (Beyotime, China). Equal protein loading was confirmed by measurement of GAPDH.

### Statistical analysis

Data are shown as means ± standard deviation, and all statistical analyses were performed using GraphPad Prism 5.0. The Students *t*-test was used for between-group comparisons. Values with *P* < 0.05 were considered to indicate statistically significant differences.

## Results

### Segmentation of the mouse epididymis

The epididymis was subdivided into ten segments (Fig. 1) by a method used previously [18]. Large epididymal regions (caput, corpus, cauda) were used in this study. We extracted the epididymis specimens carefully with the help of a dissecting microscope. Slight variations between epididymides with respect to the shape and size of specific segments were identified. However, three large regions were identified precisely and consistently.

### Localization of B7-H3 in mouse epididymis

**Fig. 2:**
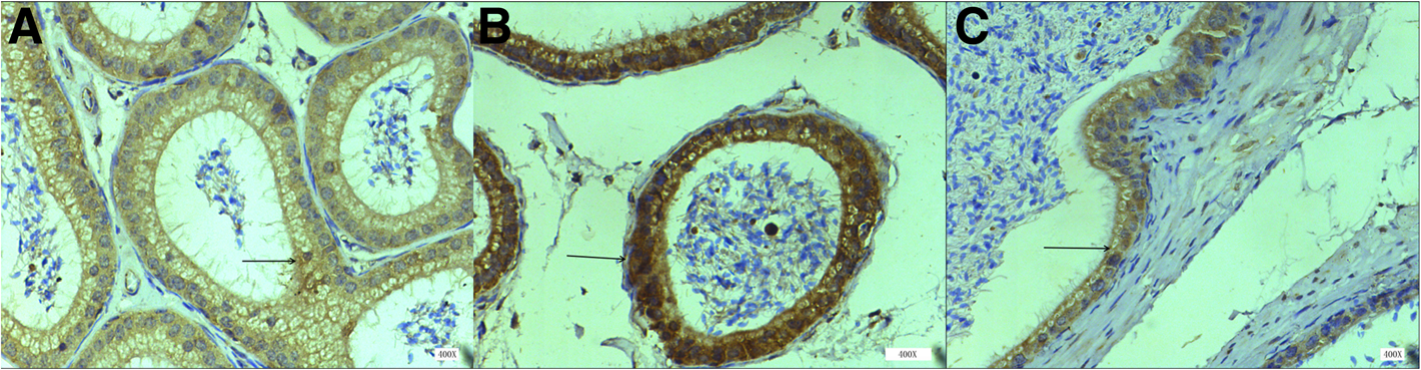
B7-H3 protein detected by Immunohistochemistry. **a** Section from the caput of the epididymis (magnification: [**a**] ×400); **b** Section from the corpus of the epididymis(magnification: [**b**] ×400); **c** Section from the cauda of the epididymis (magnification:[**c**] ×400). *Black arrows* indicate immunopositive epididymis epithelial cells

Immunohistochemical staining (Fig. 2) showed that B7-H3 staining was positive both on the membrane and in the cytoplasm of the principal cells in mouse epididymides from all regions. Moreover, the immunopositive signals of B7-H3 in the corpus were the highest of all three regions of the epididymis.

### Different levels of B7-H3 in the three regions of the epididymis

**Fig. 3:**
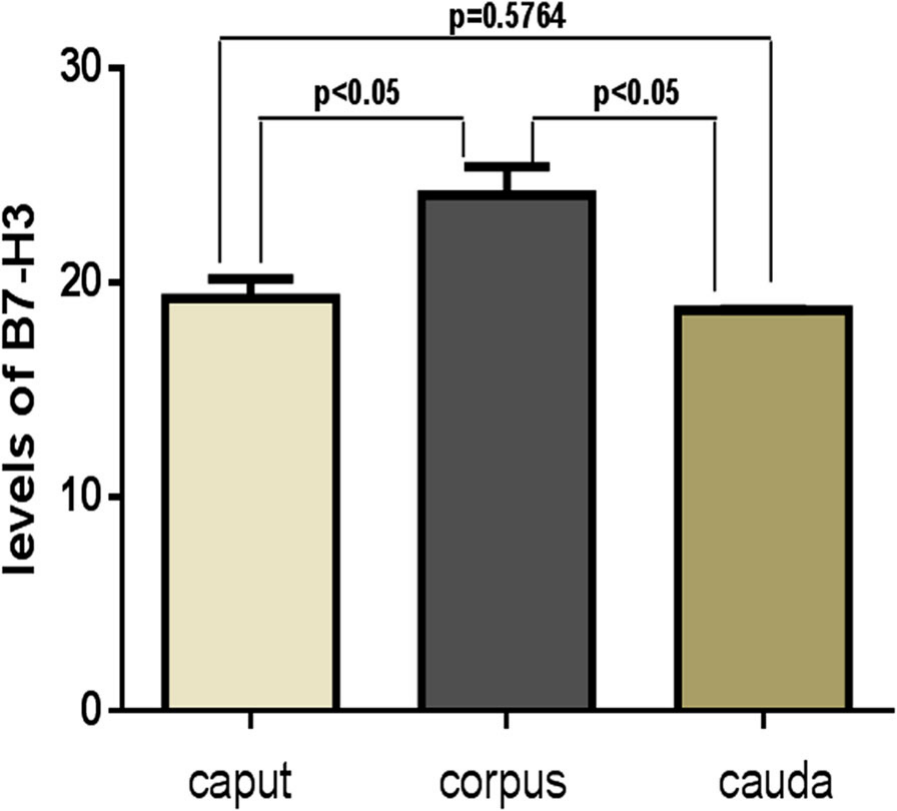
The levels of B7-H3 in the caput, corpus, cauda of the epididymis measured with ELISA. The level of B7-H3 in the corpus is higher than that in the caput (24.121 ± 2.275 versus 19.268 ± 1.583 ng/ml, *P* < 0.05) and in the cauda (24.121 ± 2.275 versus 18.712 ± 0.088 ng/ml, *P* < 0.05). The levels of B7-H3 between the caput and the cauda have no statistical difference (19.268 ± 1.583 versus 18.712 ± 0.088 ng/ml, *P* = 0.5764). All data are represented as mean ± standard deviation. Mice *n* = 12

**Fig. 4:**
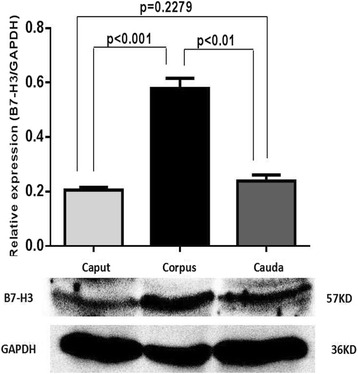
B7-H3 protein expression in the caput, corpus, cauda of the epididymis determined by western blotting. The expression of B7-H3 in the corpus is higher than that in the caput (0.578 ± 0.064 versus 0.205 ± 0.018, *P* < 0.001) and in the cauda (0.578 ± 0.064 versus 0.239 ± 0.037, *P* < 0.01). The levels of B7-H3 between the caput and the cauda have no statistical difference (0.205 ± 0.018 versus 0.239 ± 0.037, *P* = 0.2279). All data are represented as mean ± standard deviation. Mice *n* = 30

As shown in Fig. 3, the levels of B7-H3 in the caput, corpus, and cauda of the epididymis were different. The level of B7-H3 protein in the corpus of the epididymis was significantly higher than that in the caput (24.121 ± 2.275 versus 19.268 ± 1.583 ng/ml, *P* < 0.05) and in the cauda (24.121 ± 2.275 versus 18.712 ± 0.088 ng/ml, *P* < 0.05) of the epididymis. However, there was no obvious difference in the levels of B7-H3 between the caput and the cauda of the epididymis (19.268 ± 1.583 versus 18.712 ± 0.088 ng/ml, *P* = 0.5764). These results were consistent with the Western blotting data (Fig. 4).

## Discussion

At present, research on the role of B7-H3 protein has become a subject of great interest. However, most studies on B7-H3 have focused only on its immunological function. Very few studies have investigated the effects of this protein in the male reproductive field. The mammalian epididymis is the site of post-testicular sperm maturation and storage [20]. To date, large epididymal regions (caput, corpus, cauda) have been used in most studies on protein expression [18]. Each region of the epididymis contributes to the maturation, transport, concentration, or storage of sperm [21]. However, reports about the expression of B7-H3 are rare. As such, the questions of whether B7-H3 is expressed in all regions of the mouse epididymis and whether the levels of B7-H3 are different in all regions are interesting.

In this study, we examined the expression of B7-H3 in the different regions of the epididymis. Immunohistochemical staining (Fig. 2) revealed that B7-H3 protein could be detected in the caput, corpus, and cauda of the epididymis, and it was mainly located on the membrane and in the cytoplasm of the principal cells in the epididymis epithelium. Andonian et al. [22] reported that the principal cells were active in the synthesis of proteins which would be secreted into the lumen of the epididymis in a merocrine manner. It is now widely recognized that sperm maturation in the epididymis mainly results from sequential interactions with proteins secreted by the epididymal epithelium [3]. Moreover, our previous study found that seminal B7-H3 could promote sperm progressive motility and sperm concentrations are related to the seminal B7-H3 level [17]. A putative receptor for B7-H3 was also detected on the surface of sperm by our group [17]. These findings provide the basis for our hypothesis that B7-H3 can be secreted into the epididymal fluid by principal cells to contribute to epididymal sperm maturation after binding to a putative receptor on the surface of the sperm.

In addition, we determined the B7-H3 levels in the caput, corpus, and cauda of the epididymis by ELISA (Fig. 3) and western blotting (Fig. 4). Our results showed that the level of B7-H3 in the corpus of the epididymis was significantly higher than that in the caput and in the cauda of the epididymis. However, the differences in the B7-H3 levels between the caput and the cauda of the epididymis were not statistically significant. A previous study reported that the composition of the epididymal fluid protein changed continuously throughout the epididymal duct [3]. Moreover, Jelinsky et al. indicated that regional differences of the epididymis were vital to establish the luminal environment, which was required for sperm maturation [1]. As such, another hypothesis highlighted by this research is that the different levels of B7-H3 found across the three regions of the epididymis may be necessary for epididymal sperm maturation.

This study demonstrated for the first time that the levels of B7-H3 expressed in three regions of the epididymis were different. We acknowledge that our study represents preliminary results and that further experiments are needed to confirm our findings. There are also some further limitations of our study. First, all the data in this study were generated from mouse epididymides because it is difficult to obtain epididymis tissues from healthy humans. Second, despite the fact that we found that B7-H3 was expressed in all regions of mouse epididymis, the present study did not investigate its biological functions in these locations. Therefore, our future work will include exploring the effects of B7-H3 on sperm maturation in epididymis and the related mechanism.

## Conclusions

Our study demonstrated that B7-H3 was expressed in the caput, corpus, and cauda of the epididymis. However, the levels of B7-H3 expressed in three regions of the epididymis were different, which may be important for sperm maturation in the epididymis.

## Abbreviations

ELISA: Enzyme-linked immunosorbent assay
hMSCs: Human marrow stromalb cells
PBS: Phosphate–buffered saline
VEGF: Vascular endothelial growth factor
